# Phosphorylation of Ribosomal Protein S6 Kinase 1 at Thr421/Ser424 and Dephosphorylation at Thr389 Regulates SP600125-Induced Polyploidization of Megakaryocytic Cell Lines

**DOI:** 10.1371/journal.pone.0114389

**Published:** 2014-12-08

**Authors:** Chang-Ling Li, Jin-Gang Yang, Di Lin, Yong-Shan Zhao, Shuo Liu, Si-Ning Xing, Song Zhao, Cong-Qin Chen, Zhi-Ming Jiang, Fei-Fei Pu, Jian-Ping Cao, Dong-Chu Ma

**Affiliations:** 1 Department of Experimental Medicine, General Hospital of Shenyang Military Area Command, Shenhe District, Shenyang, Liaoning, China; 2 School of Life Science and Biopharmaceutics, Shenyang Pharmaceutical University, Shenhe District, Shenyang, Liaoning, China; 3 Clinical Pharmacy, Shenyang Pharmaceutical University, Shenhe District, Shenyang, Liaoning, China; University of Miami School of Medicine, United States of America

## Abstract

Megakaryocytes (MKs) are one of the few cell types that become polyploid; however, the mechanisms by which these cells are designated to become polyploid are not fully understood. In this investigation, we successfully established two relatively synchronous polyploid cell models by inducing Dami and CMK cells with SP600125. We found that SP600125 induced the polyploidization of Dami and CMK cells, concomitant with the phosphorylation of ribosomal protein S6 kinase 1 (S6K1) at Thr421/Ser424 and dephosphorylation at Thr389. The polyploidization was partially blocked by H-89, a cAMP-dependent protein kinase (PKA) inhibitor, through direct binding to S6K1, leading to dephosphorylation at Thr421/Ser424 and phosphorylation at Thr389, independent of PKA. Overexpression of a rapamycin-resistant mutant of S6K1 further enhanced the inhibitory effect of LY294002 on the SP600125-induced polyploidization of Dami and CMK cells. SP600125 also induced the polyploidization of Meg-01 cells, which are derived from a patient with chronic myelogenous leukemia, without causing a significant change in S6K1 phosphorylation. Additionally, SP600125 induced the polyploidization of HEL cells, which are derived from a patient with erythroleukemia, and phosphorylation at Thr389 of S6K1 was detected. However, the polyploidization of both Meg-01 cells and HEL cells as a result of SP600125 treatment was lower than that of SP600125-induced Dami and CMK cells, and it was not blocked by H-89 despite the increased phosphorylation of S6K1 at Thr389 in both cell lines in response to H-89. Given that the Dami and CMK cell lines were derived from patients with acute megakaryocytic leukemia (AMKL) and expressed high levels of platelet-specific antigens, our data suggested that SP600125-induced polyploidization is cell-type specific, that these cell lines were more differentiated, and that phosphorylation at Thr421/Ser424 and dephosphorylation at Thr389 of S6K1 may play an important role in the SP600125-induced polyploidization of these cell lines synergistically with other signaling pathways.

## Introduction

Megakaryocytes (MKs) are one of the few cell types that undergo a modified form of the cell cycle called endomitosis, in which cells do not undergo the late stages of mitosis and instead become polyploid [1]. This unique process is called polyploidization. It has been shown that MK endomitosis represents an incomplete multipolar mitosis characterized by a failure in both nuclear (karyokinesis) and cytoplasmic division (cytokinesis) without spindle elongation or cleavage furrow formation, producing a cell that contains a unique multilobulated nucleus [2], [3]. However, the mechanisms controlling the transition from mitosis to endomitosis are still unknown.

The mammalian target of rapamycin (mTOR) participates in the regulation of ribosome biogenesis, protein synthesis, cell growth and neurite plasticity, and it plays a critical role in metabolism, growth, proliferation and survival [4]. Thrombopoietin (TPO) induces the phosphorylation of mTOR and its effector proteins, ribosomal protein S6 kinase 1 (S6K1) and eukaryotic initiation factor 4E binding protein 1 (4E-BP1), and inhibition of the mTOR pathway by rapamycin results in a reduction in both cell proliferation and polyploidization [5], [6]. However, no significant difference was detected in the mean polyploidy level between the control culture and the treated culture when the addition of rapamycin was delayed, indicating that the effect of rapamycin on polyploidization may be indirect or mediated by the inhibition of the G1/S transition in proliferative progenitors [5], [6]. Moreover, the role of S6K1 and 4E-BP1 in the polyploidization of MKs is not clearly understood. Previously, we demonstrated that S6K1 was involved in polyploidization through its phosphorylation at Thr421/Ser424 during M phase in nocodazole-treated Dami cells [7]. However, in nocodazole-induced polyploidization, the cells are multinucleated (karyokinesis is not affected), while in MKs, the nuclei are polylobulated.

In this study, Dami, CMK, Meg-01 and HEL cells were treated with SP600125. Relatively synchronized polyploid cell models with polylobulated nuclei were established from Dami and CMK cells, and these cells were more similar to primary MKs that have undergone physiological polyploidization than polyploid cells induced by nocodazole. With these models, we found that SP600125 induced polyploidization of more differentiated cell lines through the phosphorylation of S6K1 at Thr421/Ser424 and dephosphorylation at Thr389 of S6K1 synergistically with other signaling pathways.

## Materials and Methods

### Reagents

Dimethylsulfoxide (DMSO) and nocodazole were purchased from Sigma (St. Louis, MO). SP600125 and H-89 were purchased from LC Laboratories (Woburn, MA). PD184352, U0126 and LY294002 were purchased from Selleckchem (Munich, Germany). SuperSignal West Pico Chemiluminescent Substrate was purchased from Pierce Biotechnology (Rocklord, IL).

### Antibodies

S6K1 antibody, phospho-S6K1 (Thr389) antibody, phospho-S6K1 (Thr421/Ser424) antibody, 4E-BP1 antibody, phospho-4E-BP1(Thr37/46) antibody, phospho-4E-BP1 (Ser65) antibody, phospho-4E-BP1 (Thr70) antibody, eIF4E antibody, phospho-eIF4E (Ser209) antibody, p44/42 MAP kinase antibody, phospho-p44/42 MAP kinase (Thr202/Tyr204) antibody, phospho-Akt (Ser473) antibody, Akt antibody, phospho-PKA substrate (RRXS*/T*) (100G7E) antibody, phospho-(Ser/Thr) PKA substrate antibody, c-Myc antibody, cyclin B1 antibody, survivin antibody, β-actin antibody, and goat-rabbit IgG conjugated with HRP were purchased from Cell Signaling Technology. Cyclin D3 rabbit polyclonal antibody was purchased from Santa Cruz Biotechnology, Inc. (Santa Cruz, CA). CD3 antibody (FITC-conjugated), CD13 antibody (PE conjugated), CD19 antibody (perCP conjugated), CD33 antibody (PE conjugated), CD34 antibody (PE-conjugated), CD41a antibody (PE-conjugated), CD42a antibody (FITC conjugated), CD42b antibody (FITC conjugated), CD71 antibody (FITC conjugated) and CD235a (FITC conjugated) were purchased from Becton Dickinson (BD).

### Cell lines and culture conditions

Dami, HEL and Meg-01 cells were purchased from ATCC (ATCC number: CRL-9792, TIB-180 and CRL-2021, respectively) and grown in RPMI 1640 medium (Life Technologies, Grand Island, NY) containing 10% fetal calf serum (FCS) (Zhejiang Tianhang Biological Technology Co., Ltd., Hangzhou, Zhejiang, China) for Dami cells or fetal bovine serum (FBS) (Hyclone, Logan, UT) for HEL and Meg-01 cells. CMK cells were obtained from the German Collection of Microorganisms and Cell Cultures (DSMZ; Braunschweig, Germany) and cultured in RPMI 1640 medium with 10% FCS. The cultures were maintained in a humidified atmosphere of 5% CO_2_ at 37°C. To determine the growth curve, the cells were seeded at 2×10^5^/ml in 10 ml of complete media and cultured in 25-cm^2^ tissue culture flasks (Costar, Corning Incorporated, Corning, NY). Cell counts were performed using the trypan blue dye exclusion method.

### Treatment of cells

Cells were seeded at 2×10^5^/ml and treated with SP600125 at different concentrations for the times indicated. In some experiments, 1 hour prior to the addition of SP600125, H-89 was added to the culture at different concentrations as indicated. In other experiments, 1 hour prior to the addition of SP600125, PD184352 (2 µM), U0126 (10 µM), or LY294002 (30 µM) was added to the culture separately (see below). In all experiments, the final concentration of DMSO was adjusted to 0.1% as a vehicle control. After incubation, the treated or untreated cells were separately collected and washed with 3 ml PBS three times. The cell number and viability were determined using a hemocytometer and the trypan blue dye exclusion method. Cells were fixed with 80% methanol for flow cytometric analysis, cytocentrifuged onto glass slides for morphological analysis, or lysed for western blot analysis (see below).

### Phenotypic analysis

Dami, CMK, Meg-01 and HEL cells were labeled with CD3, CD13, CD19, CD33, CD34, CD41a, CD42a, CD42b, and CD71, CD235a. Different isotypic antibodies were used as controls. The labeled cells were analyzed with a FACSCanto II flow cytometer (Becton Dickinson).

### Morphological analysis

Cells collected on different days of culture were cytocentrifuged onto glass slides, stained with Wright-Giemsa and then identified by morphological analysis.

### Western blot analysis

Western blot analysis was performed as described previously [7] with slight modifications. Briefly, the collected cells were washed with PBS and then lysed with sample buffer containing protease inhibitor cocktail tablets (cOmplete ULTRA Tablets, Roche Applied Science, Indianapolis, IN) according to the manufacturer’s guidelines. The protein concentration was measured by means of the BCA protein assay reagent kit (PIERCE Biotechnology, Rockford, IL) to ensure equal electrophoretic loading. The lysates were loaded onto 6–15% SDS–PAGE gels and transferred to nitrocellulose membranes. The blots were blocked using 5% non-fat milk in Tris-buffered saline containing 0.1% Tween-20 (TBST) for 2 h at room temperature. The membranes were incubated overnight at 4°C with different primary antibodies. After three washes in TBST, the membranes were incubated with goat anti-rabbit IgG conjugated to HRP at room temperature for 2 h. The reactive proteins were detected using the SuperSignal West Pico Chemiluminescent Substrate, and the luminograms were prepared by exposing the immunoblots to X-ray film. The density of the protein bands was analyzed with Image J.

### Ploidy analysis

Cells fixed with 80% methanol were maintained for at least 24 h at −20°C and washed in PBS containing 1% BSA and then permeabilized with 0.25% Triton X-100 at 4°C for 15 min. DNA was stained with PBS containing 50 µg/ml propidium iodide (PI) and 100 µg/ml RNAse A (Sigma) for 2 h. The cells were analyzed on a FACSort flow cytometer (Becton Dickinson). For each sample, 10000 cells were acquired. The data were analyzed using the CellQuest software package as described previously [8].

### Molecular docking study

The initial crystal structures of S6K1 (PDB ID: 3A61 and 3A62) were retrieved from the Protein Data Bank (http://www.rcsb.org/pdb). The data for H-89 were downloaded from PubChem, and energy minimization was performed until an energy gradient of 0.001 kcal mol Å^−1^ was reached. To investigate the interactions between H-89 and the protein kinases, AutoDock 4.2 software was used to perform the docking calculations. The graphical user interface program AutoDockTools was used to add polar hydrogens and partial charges for protein using the Kollman United Atom charges. Atomic solvation parameters and fragmental volumes for the proteins were assigned using the addsol utility under docking simulations. The Gasteiger charge was assigned, and the non-polar hydrogens were merged onto the structure of H-89.

The docking procedure was applied to the whole-protein target, without imposing the binding site (“blind docking”). A 120×120×126 Å grid box with a grid spacing of 0.375 Å was generated for protein kinases; this grid box was large enough to cover the entire surface of each protein. Affinity grid fields were generated using the auxiliary program AutoGrid4.2. The Lamarckian genetic algorithm (LGA) was used to find the appropriate binding positions, orientations, and conformations of the ligands. The default parameters were used, except for the number of generations, which was set at 100. The resulting data were obtained from docking experiments in which the lowest energy conformation in the largest cluster of each docking simulation was extracted and analyzed. The hydrogen bonds and hydrophobic interactions between H-89 and the protein kinases were represented with Ligplot.

### S6 Kinase Activity Assay

The in vitro effect of H-89 on the S6 kinase was tested using the p70 S6K activity kit (Enzo life science) according to the manufacturer’s protocol with slight modification. Briefly, the appropriate quantity of p70 S6K was incubated with different concentrations of H-89 for 10 minutes. After incubation, the mixture was added to a microtiter plate coated with the substrate peptide for p70 S6K, followed by the addition of ATP to initiate the reaction. After terminating the kinase reaction by emptying the contents of each well, a phosphospecific substrate antibody was added. The bound phosphospecific antibodies were detected with a peroxidase-conjugated secondary antibody and visualized by adding tetramethylbenzidine (TMB) substrate after stopping the color development by adding an acid stop solution. The intensity of the color was measured at 450 nm using a Synergy™ 2 Multi-Mode Microplate Reader (BioTek). The maximal kinase activity and the percentage of maximal activity were calculated using the following two formulas: kinase activity = (average absorbance (sample)-average absorbance (blank))/quantity of purified kinase used per assay, and percentage of maximal activity (%) = kinase activity (sample)/kinase activity (positive control)×100%.

### Site-directed mutagenesis

Site-directed mutagenesis was carried out using the standard polymerase chain reaction with mutated pairs of primers and pfu DNA polymerase (SK2084, Sangon Biotech (Shanghai) Co., Ltd.). Multi-site-directed mutagenesis was carried out by mutating one amino acid at a time. The pRK7-HA-S6K1-WT plasmids were obtained from Addgene (deposited by John Blenis [9]) and were used as the template. After digestion with DpnI (D1233A, Takara, Japan), the mutated plasmids were transformed into E. coli DH5α and screened on plates with ampicillin. After selective screening for positive clones, the isolated plasmid DNA was sequenced to confirm that the base substitutions were correct and that no other unwanted mutations had been introduced. All mutants used are shown in Table 1.

**Table 1:** Plasmids containing S6K1 mutants generated by site-directed mutagenesis.

Site of mutagenesis	Designation	Abbreviation
None	pRK7-HA-S6K1-WT	S6K1-WT
Thr389→Glu (T→E)	pRK7-HA-S6K1-T389E	S6K1-T389E
Thr389→Ala (T→A)	pRK7-HA-S6K1-T389A	S6K1-T389A
Ser411→Asp (S→D)	pRK7-HA-S6K1-D3E	S6K1-D3E
Ser418→Asp (S→D)		
Thr421→Glu (T→E)		
Ser424→Asp (S→D)		

None: No mutant was created.

### Nucleofections

Dami or CMK cells were transiently transfected with plasmids encoding S6K1-WT or the S6K1-T389E, S6K1-T389A and S6K1-D3E mutants as described elsewhere [7]. Briefly, Dami or CMK cells were washed in cold phosphate-buffered saline and resuspended in the specified electroporation buffer to a final concentration of 1.2×10^8^ cells/ml. Two micrograms of each of the mutant plasmids (described above) was mixed with 0.1 ml of cell suspension. The mixture was transferred to a 2.0-mm electroporation cuvette and nucleofected with an Amaxa Nucleofector™ apparatus (Amaxa, Cologne, Germany). After transfection, the cells were resuspended in RPMI 1640 medium with 10% FCS and incubated overnight. After incubation, the transfected Dami or CMK cells were induced with SP600125 (32 µM) for 72 hours in a humidified atmosphere of 5% CO_2_ at 37°C after pretreatment with or without U0126 (10 µM) and LY294002 (30 µM) for 1 hour. Dami or CMK cells that were not transfected with mutant plasmids but were induced with SP600125 were used as controls. After a 72-hour incubation, the cells were fixed and prepared for flow cytometric analysis as described above.

### Statistical Analysis

The data are represented as the mean ± SEM from at least three independent experiments. The statistical significance was calculated with Student’s t test: *p<0.05, **p<0.01.

## Results

### SP600125 induced polyploidization in a dose- and time-dependent manner

Previous studies revealed that the c-Jun N-terminal kinase (JNK) inhibitor SP600125 induced polyploidization in some cell lines [10], [11], [12]. Therefore, SP600125 was used to treat four different cell lines: Dami and CMK cells, which are AMKL cell lines, Meg-01 cells, which is a leukemic cell line derived from a patient with chronic myelogenous leukemia with a megakaryocytic phenotype [13], [14], [15], and HEL cells, which is a cell line with significant similarity to MKs that was derived from an erythroleukemia patient (Fig. S1) [16].

**Figure 1:**
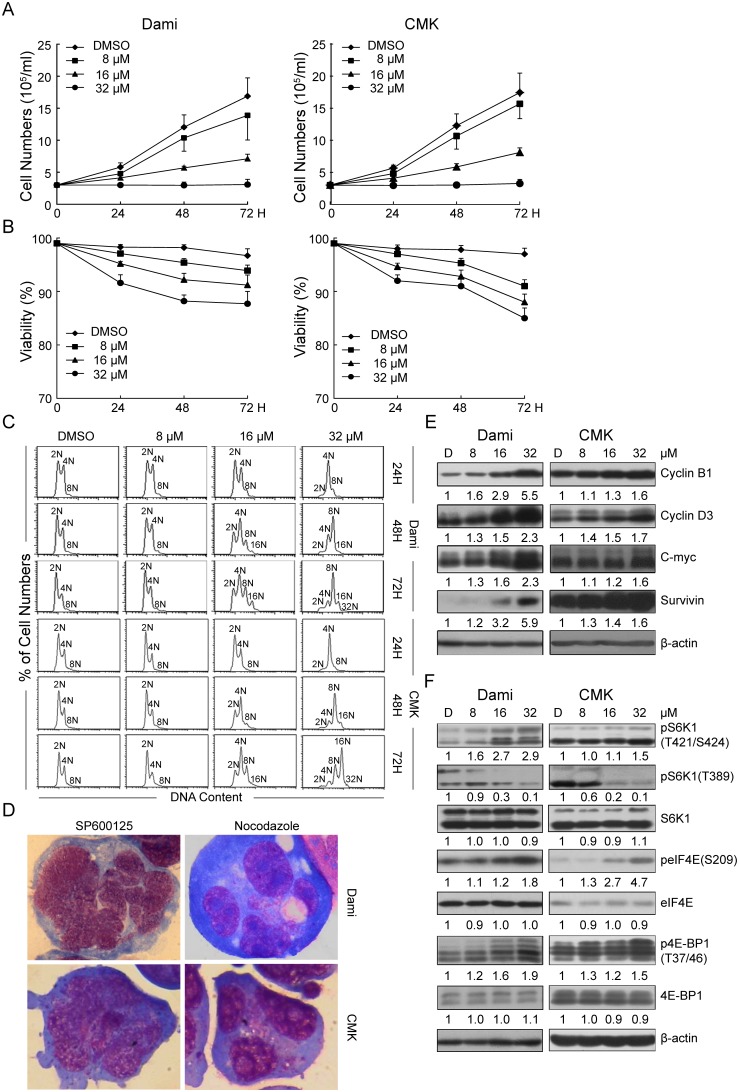
Effect of SP600125 on proliferation, viability, polyploidy, expression of cyclin B1, cyclin D3, c-Myc, and survivin, and the translation-related proteins of Dami and CMK cells. Dami and CMK cells were seeded at 2×10^5^/ml in RPMI 1640 medium containing 10% FCS and treated with SP600125 at different concentrations for different periods of time as indicated. Dami and CMK cells treated with DMSO were used as a control. (A, B) The cell number and viability, presented as the mean±SEM, were determined from 4 separate experiments. (C) Representative DNA histograms of SP600125-induced Dami and CMK cells analyzed by flow cytometry. (D) Morphology was analyzed by Wright-Giemsa staining of cytocentrifuged preparations of Dami and CMK cells induced by SP600125 or nocodazole (Original magnification, 1000×). The Dami and CMK cells treated with DMSO or with SP600125 were lysed, and equal amounts of protein were loaded for western blots to evaluate the protein levels of cyclin B1, cyclin D3, c-Myc, and survivin (E), and the phosphorylation and protein levels of S6K1, eIF4E and 4E-BP1(F). β-actin was used as an internal control.

SP600125 inhibited the growth of Dami, CMK, Meg-01 and HEL cells in a dose- and time-dependent manner. However, the effect of SP600125 on the viability differed among the cell lines. The viability of Dami and CMK cells was slightly affected by SP600125 at concentrations up to 32 µM after a 72-hour induction, and the viability of the other two cell lines was significantly affected, particularly HEL cells. Only 50% of HEL cells were found to exclude trypan blue staining after 72-hours of treatment with SP600125 at 32 µM (Fig. 1A and B; Fig. S2A and B). Therefore, in the subsequent studies, Dami, CMK, and Meg-01 cells were treated with 32 µM SP600125, and HEL cells were treated with 24 µM SP600125.

The analysis of polyploidy by flow cytometry revealed that SP600125 induced the polyploidization of Dami, CMK and HEL cells in a dose- and time-dependent manner (Fig. 1C; Fig. S2C). However, SP600125 only increased the frequency of tetraploidy (4N) in Meg-01 cells (Fig. S2C). Morphologic analysis revealed that the nuclei of SP600125-induced polyploid Dami and CMK cells were polylobulated, and the cells contained less cytoplasm, whereas nocodazole-induced polyploid cells were multinucleated (Fig. 1D). Similar data were obtained from HEL cells (data not shown). However, Meg-01 cells with higher ploidy induced by nocodazole or SP600125 had single nuclei (Fig. S2D). The Dami, CMK and HEL cells expressed high levels of platelet-specific antigens (Fig. S1), indicating that SP600125-induced polyploidization in these more differentiated cell lines more closely resembled the physiological polyploidization of primary MKs than that induced by nocodazole.

Cyclin B1, cyclin D3, c-Myc, and survivin are involved in regulating the polyploidization of MKs [17], [18], [19], [20], [21], [22]. As shown in Fig. 1E, SP600125 increased the expression of cyclin B1, cyclin D3, c-Myc, and survivin in a dose-dependent manner in Dami and CMK cells. However, only cyclin B1 and survivin were enhanced in SP600125-induced HEL cells (Fig. S2E). Moreover, the expression of cyclin B1, cyclin D3 and survivin in SP600125-induced Meg-01 cells was not affected, while the expression of c-Myc (Fig. S2E) decreased slightly. Although SP600125 increased the phosphorylation of 4E-BP1 at Thr37/46 in Dami, CMK, Meg-01 and HEL cells, the phosphorylation of eIF4E at Ser209 was only observed in Dami and CMK cells (Fig. 1F; Fig. S2F). The phosphorylation of 4E-BP1 at Thr37/46 primes the subsequent phosphorylation at Ser65 and Thr70, which decreases the affinity of 4E-BP1 for eIF4E, allowing eIF4G and its associated factors to bind to eIF4E and promote the assembly of the translation preinitiation complex [23]. Furthermore, the phosphorylation of eIF4E at Ser209 increases the affinity of eIF4E for capped mRNA [24], [25]. Considering these findings, our data indicated that SP600125 promoted translation while inducing polyploidization in Dami and CMK cells. Interestingly, SP600125 decreased the phosphorylation of S6K1 at Thr389 and increased the phosphorylation of S6K1 at Thr421/Ser424 in Dami and CMK cells (Fig. 1F). These alterations of S6K1 and 4E-BP1 are consistent with our data previously obtained in a nocodazole-induced model of polyploidy based on Dami cells [7]. In contrast, SP600125 only increased the phosphorylation of Thr389 (Fig. S2F) in HEL cells, although it did not significantly affect the phosphorylation of either Thr389 or Thr421/Ser424 in Meg-01 cells (S2F). Taken together, these data suggest that the regulatory effects of SP600125 on S6K1 and 4E-BP1 in SP600125-induced polyploid cells may promote translation and are cell type-specific. Furthermore, the dephosphorylation of S6K1 at Thr389 may play an important role in SP600125-induced polyploidization.

### H-89 blocked SP600125-induced polyploidization by changing the phosphorylation state of S6K1

**Figure 2:**
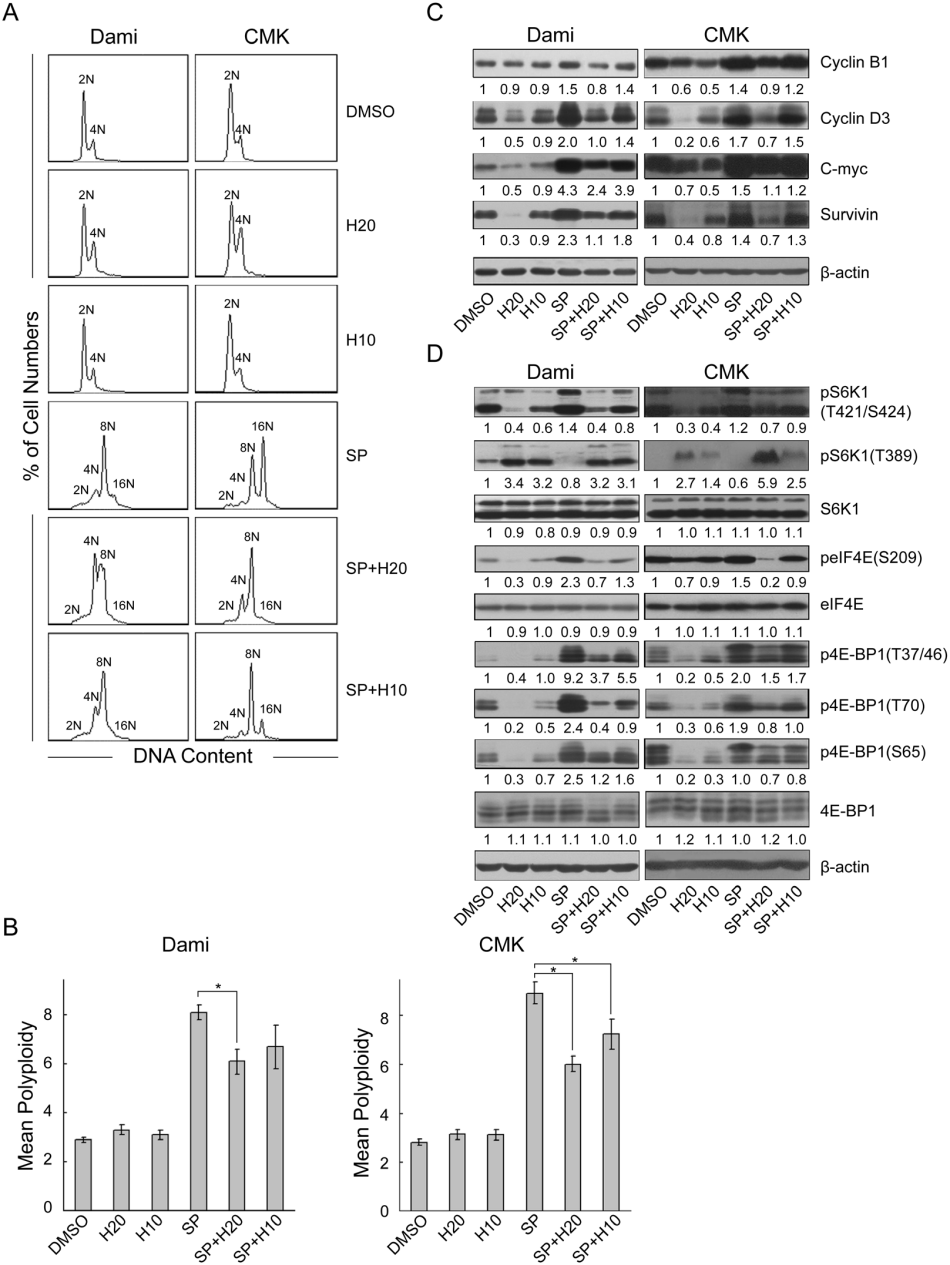
H-89 blocked polyploidization of the SP600125 treated-Dami and CMK cells. Dami and CMK cells were treated with SP600125 at 32 µM for 72 hours after pretreatment without or with H-89 at 10 µM or 20 µM for 1 hour. Dami and CMK cells treated with DMSO were used as the vehicle-treated control, and Dami or CMK cells treated with H-89 alone were used as the pretreatment control. After incubation, the cells were fixed, stained with PI and analyzed with a flow cytometer for DNA ploidy (A). The data are presented as the mean±SEM polyploidy and were obtained from 4 separate experiments (B). All bar graphs depict the means ± SEM; *p<0.05. The cells were lysed, and equal amounts of protein were analyzed by western blot for cyclin B1, cyclin D3, c-Myc, and survivin (C). The phosphorylation and protein levels of S6K1, eIF4E and 4E-BP1 (D). β-actin was used as an internal control.

S6K1 and cAMP-dependent protein kinase (PKA) are members of the AGC kinase family, and PKA can phosphorylate eEF2K, which participates in translation elongation [26], [27], [28]. We speculated that the inhibition of translation would affect the polyploidization of MKs. Therefore, we further investigated if H-89, a PKA inhibitor with a strong inhibitory effect on AGC kinases [29], [30], could block the polyploidization induced by SP600125. As expected, the polyploidization of SP600125-induced Dami and CMK cells was partially blocked by H-89 (Fig. 2A and B). However, H-89 did not inhibit the polyploidization of SP600125-induced Meg-01 cells (Fig. S3A and B). Surprisingly, H-89 promoted the polyploidization of SP600125-induced HEL cells. H-89 had no significant effect on the DNA distribution when it was used alone in any of the cell lines tested.

Western blot analysis demonstrated that H-89 decreased the expression of cyclin B1, cyclin D3, survivin and c-Myc in the SP600125-induced polyploidy Dami and CMK cells while it blocked polyploidization (Fig. 2C). As expected, H-89 decreased the phosphorylation of 4E-BP1 at Thr37/46, Ser65 and Thr70 and the phosphorylation of eIF4E at Ser209 in SP600125-induced-Dami and CMK cells (Fig. 2D). Moreover, H-89 decreased the phosphorylation of S6K1 at Thr421/Ser424 and increased the phosphorylation of S6K1 at Thr389 in SP600125-induced polyploid Dami and CMK cells. In addition, H-89 decreased the phosphorylation of eIF4E in SP600125-induced polyploid Dami and CMK cells. By contrast, H-89 increased the phosphorylation of S6K1 at Thr389 without significantly affecting Thr421/Ser424 in SP600125-induced Meg-01 and HEL cells (Fig. S3D). In HEL cells, H-89 further increased the phosphorylation of S6K1 at Thr389, compared to the phosphorylation level of Thr389 induced by SP600125 alone. However, H-89 decreased the phosphorylation of 4E-BP1 in both SP600125-induced Meg-01 and HEL cells. Moreover, H-89 had no significant effect on the regulation of cyclin B1, cyclin D3, survivin and c-Myc in SP600125-induced Meg-01 and HEL cells compared with the effect of H-89 on SP600125-induced Dami and CMK cells. Therefore, these data indicated that phosphorylation at Thr421/Ser424 and dephosphorylation at Thr389 of S6K1 may play an important role in SP600125-induced polyploidization. In addition, CMK cells, which were derived from a Down’s syndrome patient suffering from AMKL, expressed high levels of CD41a (72.1%), and Dami cells, which were derived from an AMKL patient, expressed high levels of CD41a (64.2%), CD42a (89.8%), and CD42b (66.1%); these cells represent more differentiated megakaryocytic cell lines compared with HEL and Meg-01 cells (Fig. S1) [13], [14]. Meg-01 cells, which were derived from a patient with blast crisis of Philadelphia (Ph1) chromosome-positive chronic myelogenous leukemia, expressed low levels of CD41a (8.9%) and represent a less differentiated megakaryocytic cell line (Fig. S1) [15]. Finally, HEL cells, which were established from a patient with erythroleukemia, represent a less differentiated megakaryocytic cell line, although they expressed high levels of CD41a (59.1%) (Fig. S1) [16]. Taken together, these data revealed that H-89 inhibited SP600125-induced polyploidization in more differentiated cell lines (Dami and CMK cells) through dephosphorylation at Thr421/Ser424 and phosphorylation at Thr389 of S6K1. These alterations may down regulate the protein translation of cyclin B1, cyclin D3, survivin and c-Myc.

### H-89 altered the phosphorylation state of S6K1 through direct binding

To investigate whether H-89 altered the phosphorylation state of S6K1 directly or indirectly, we next performed docking studies to investigate whether H-89 binds to S6K1. Recently, Sunami et al. reported the crystal structures of the kinase domain of S6K1 bound to staurosporine in both the unphosphorylated state and in the 3′-phosphoinositide-dependent kinase-1-phosphorylated state in which Thr252 (Thr229 of p70-S6K1) of the activation loop is phosphorylated [31]. Unphosphorylated S6K1 exists in two crystal forms; in one of these, the S6K1 kinase domain exists as a monomer (Form II), and in the other, it exists as a domain-swapped dimer (Form I). However, domain swapping has been observed to be an artifact in a number of proteins in which the isolated domains were dimers [31]. Therefore, we used the crystal structures of phosphorylated S6K1 (PDB: 3A62) and unphosphorylated S6K1 (PDB: 3A61) to perform the docking studies as described in Material and Methods.

**Figure 3:**
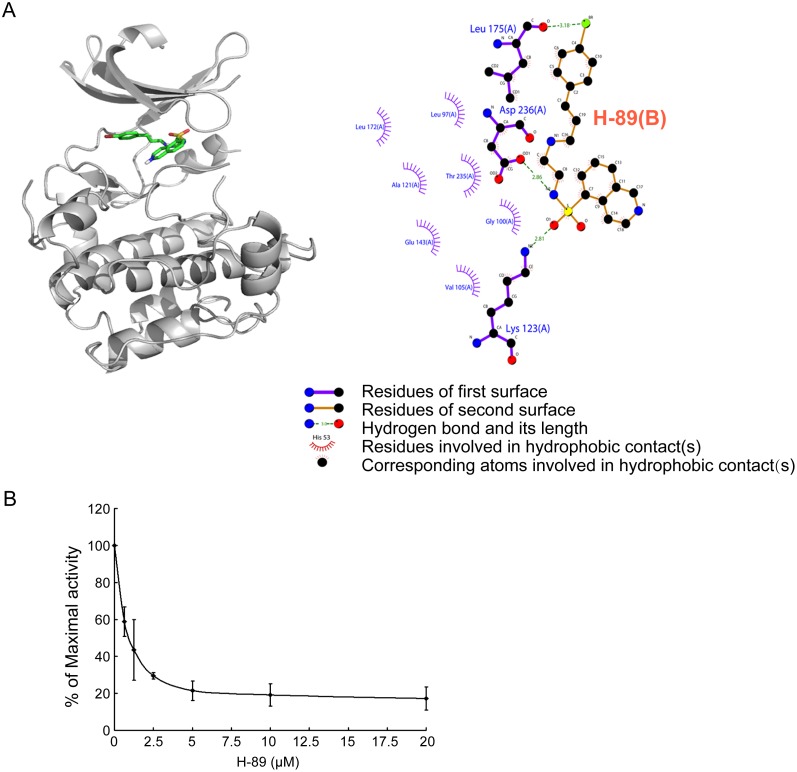
H-89 inhibited the activity of S6K1 directly. (A) Docking studies were performed to evaluate the binding of H-89 to S6K1 using AutoDock 4.2 software. H-89 is predicted to bind into the hydrophobic cleft between the N- and C-terminal domains of unphosphorylated S6K1 (PDB: 3A61). (B) The activity of S6K1 was assayed in vitro in the presence of H-89 at increasing concentrations as indicated. The data are presented as the mean±SEM of the percentage of kinase activity relative to the control measured in the presence of DMSO and were obtained from 3 separate experiments.

As shown in Figure 3A, H-89 was predicted to bind to the ATP binding pocket situated in the hydrophobic cleft between the N- and C-terminal domains of unphosphorylated S6K1. This prediction was also obtained with the phosphorylated S6K1 (Fig. S4). Therefore, we further investigated the effect of H-89 on the activity of S6K1 kinase in vitro by using recombinant p70 S6K kinase. As shown in Figure 3B, H-89 inhibited p70 S6K activity in vitro over the range of tested concentrations (0.625 µM, 1.25 µM, 2.5 µM, 5 µM, 10 µM, and 20 µM) in a dose-dependent manner with an IC_50_ of 1.9 µM. These data indicated that H-89 bonded directly to S6K1 and inhibited its activity.

### H-89 blocked SP600125-induced polyploidization independently of its effect on PKA activity

**Figure 4:**
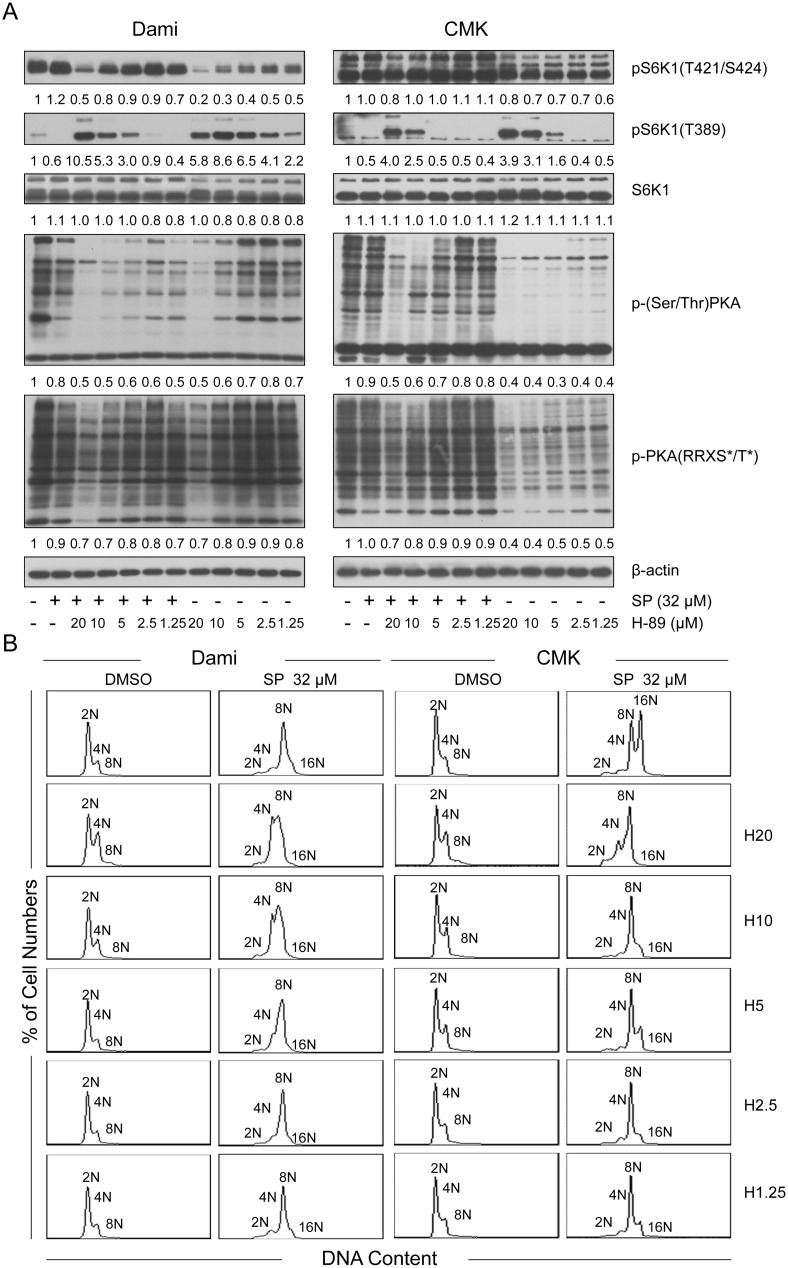
H-89 blocked the polyploidization of Dami and CMK cells independent of PKA. Dami and CMK cells were treated with SP600125 at 32 µM for 72 hours after pretreatment with or without H-89 at increasing concentrations as indicated for 1 hour. Dami or CMK cells treated with DMSO were used as the vehicle-treated control, and Dami or CMK cells treated with H-89 alone were used as the pretreatment control. (A) The cells were lysed, and equal amounts of protein were analyzed by western blot for Phospho-PKA Substrate (RRXS*/T*), Phospho-(Ser/Thr) PKA Substrate, S6K1, phospho-S6K1 (Thr421/Ser424), and phospho-S6K1 (Thr389). (B) The cells were fixed, stained with PI and analyzed with a flow cytometer for DNA ploidy.

Because H-89 is a PKA inhibitor, we further investigated the effect of SP600125 on PKA and the role of PKA in SP600125-induced polyploidization of Dami and CMK cells by using phospho-PKA substrate antibodies. As shown in Figure 4A, SP600125 slightly decreased the phosphorylation of PKA substrates in Dami and CMK cells while inducing polyploidization. As expected, H-89 further decreased the phosphorylation of the PKA substrates while blocking the SP600125-induced polyploidization of Dami and CMK cells in a dose-dependent manner (Fig. 4B). However, H-89 increased the phosphorylation of S6K1 at Thr389 and decreased the phosphorylation at Thr421/Ser424 while blocking the polyploidization of both SP600125-induced Dami and CMK cells in a dose-dependent manner. In contrast, H-89 further decreased the phosphorylation of the PKA substrates and increased the phosphorylation of S6K1 at Thr389, which did not lead to the inhibition of SP600125-induced polyploidization in Meg-01 cells (Fig. S5, Fig. S4A and B). Taken together, these results demonstrate that H-89 blocked the SP600125-induced polyploidization of Dami and CMK cells mainly by producing a specific S6K1 phosphorylation state, independently of its inhibitory effect on PKA.

### Partial inhibition of S6K1 phosphorylation at Thr421/Ser424 is not sufficient to inhibit the SP600125-induced polyploidization of Dami and CMK cells

It was previously reported that the proline-directed mitogen-regulated MAPKs phosphorylate Thr421/Ser424, which lie in the C-terminal autoinhibitory pseudosubstrate domain of S6K1 [32]. In addition, Akt activates S6K1 via mammalian target of rapamycin complex 1 (mTORC1) [33]. However, the physiological kinases for these sites in intact cells are unknown. Therefore, we used to investigate whether these pathways are involved in the phosphorylation of S6K1 in SP600125-induced polyploid Dami and CMK cells using PD184352 (a potent and selective MEK1/2 inhibitor), U0126 (a MEK inhibitor), and LY294002 (a PI3K inhibitor). PD184352, U0126, and LY294002 were used at concentrations of 2 µM, 10 µM, and 30 µM, respectively. These concentrations were chosen based on inhibition of their substrates (data not shown). When used alone, these inhibitors had no effect on the DNA content distribution of Dami and CMK cells (data not shown).

**Figure 5:**
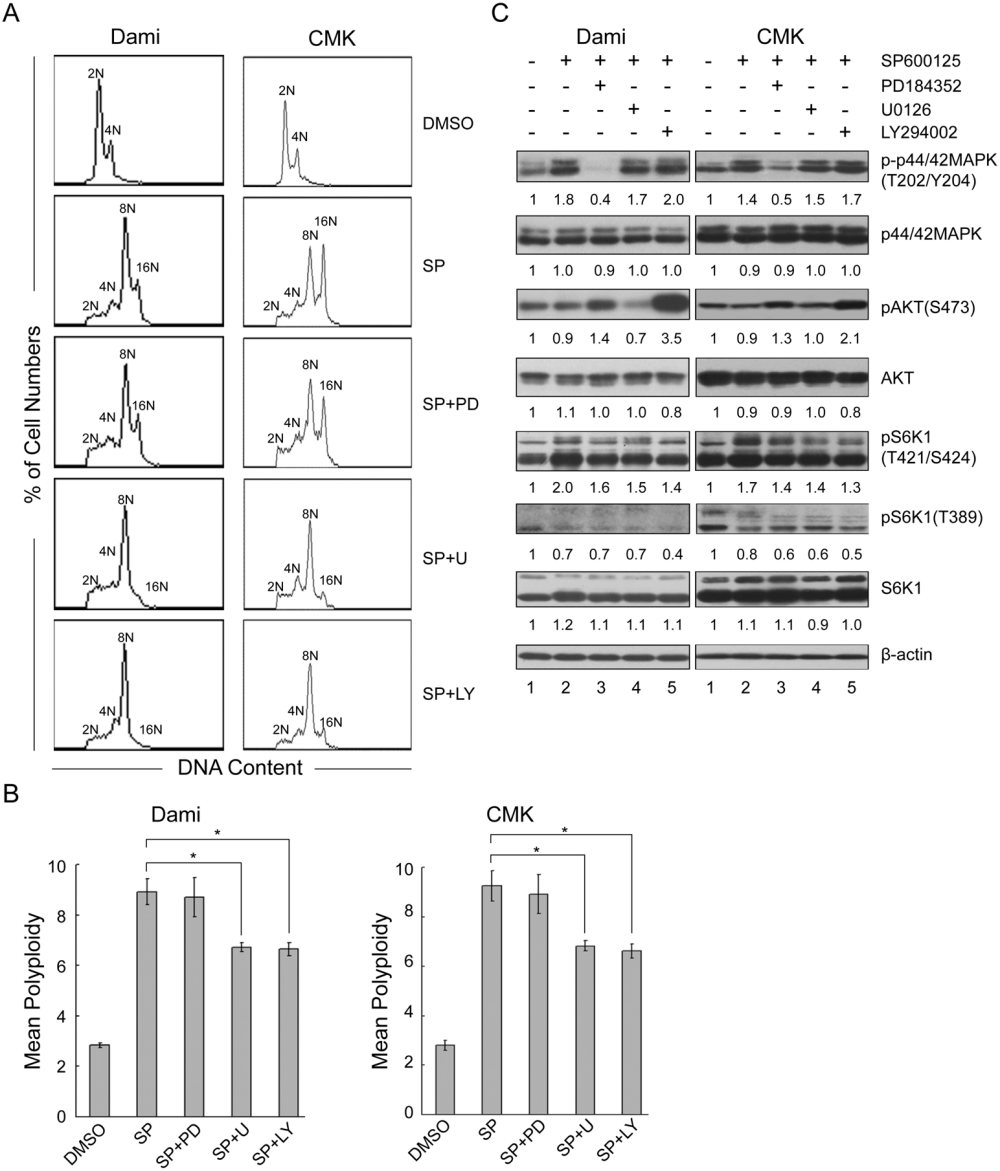
Partial inhibition of phosphorylation of S6K1 at Thr421/Ser424 is not sufficient to block polyploidization. Dami and CMK cells were incubated with SP600125 at 32 µM for 72 hours after pretreatment with or without PD184352 (2 µM), U0126 (10 µM), or LY294002 (30 µM) for 1 hour. Dami and CMK cells treated with DMSO were used as the control. After incubation, the cells were fixed, stained with PI and analyzed with a flow cytometer for DNA ploidy. (A) A typical representative DNA histogram. (B) The data are presented as the mean±SEM level of polyploidy and were obtained from 4 separate experiments; *p<0.05. (C) The remaining cells were lysed, and equal amounts of protein were analyzed by western blot for p44/42 MAPK, phospho-p44/42 MAPK (Thr202/Tyr204), Akt, phospho-Akt (Ser473), S6K1, phospho-S6K1 (Thr421/Ser424), and phospho-S6K1 (Thr389). β-actin was used as an internal control.

PD184352 had no significant effect on the ploidy in SP600125-induced Dami and CMK cells (Fig. 5A and B), although it significantly blocked the phosphorylation of p44/42 MAPK at Thr202/Tyr204, which was markedly enhanced by SP600125 (Fig. 5C, lane 2). Surprisingly, U0126 blocked the SP600125-induced polyploidization of Dami and CMK cells (Fig. 5A) and reduced the mean polyploidy (Fig. 5B). However, U0126 had no significant effect on the phosphorylation of p44/42 MAPK at Thr202/Tyr204 (Fig. 5C, lane 4). These data indicate that SP600125 induced the polyploidization of Dami and CMK cells independently of MAPK and that U0126 blocked the polyploidization through other signaling pathways.

Similarly to U0126, LY294002 also blocked the SP600125-induced polyploidization of Dami and CMK cells (Fig. 5A and B). Unexpectedly, although LY294002 inhibited the phosphorylation of Akt at Ser473 in the untreated Dami and CMK cells (data not shown), it markedly increased the phosphorylation of Akt in SP600125-induced polyploid Dami and CMK cells (Fig. 5C, lane 5). This result indicated that the PI3K/Akt pathway was activated in SP600126-induced polyploid Dami and CMK cells pretreated with LY294002. Notably, PD184352, U0126, and LY294002 partially inhibited the phosphorylation of S6K1 at Thr421/Ser424 in SP600125-induced polyploid Dami and CMK cells. However, these inhibitors did not increase the phosphorylation of S6K1 at Thr389. Taken together, these data suggest that the phosphorylation of S6K1 at Thr421/Ser424 alone may not be sufficient to mediate the polyploidization of Dami and CMK cells induced by SP600125, and other signaling pathways may be involved in this process, independent of Akt.

### The C-terminal autoinhibitory pseudosubstrate domain in S6K1 is involved in the regulation of SP600125-induced polyploidization of Dami and CMK cells

**Figure 6:**
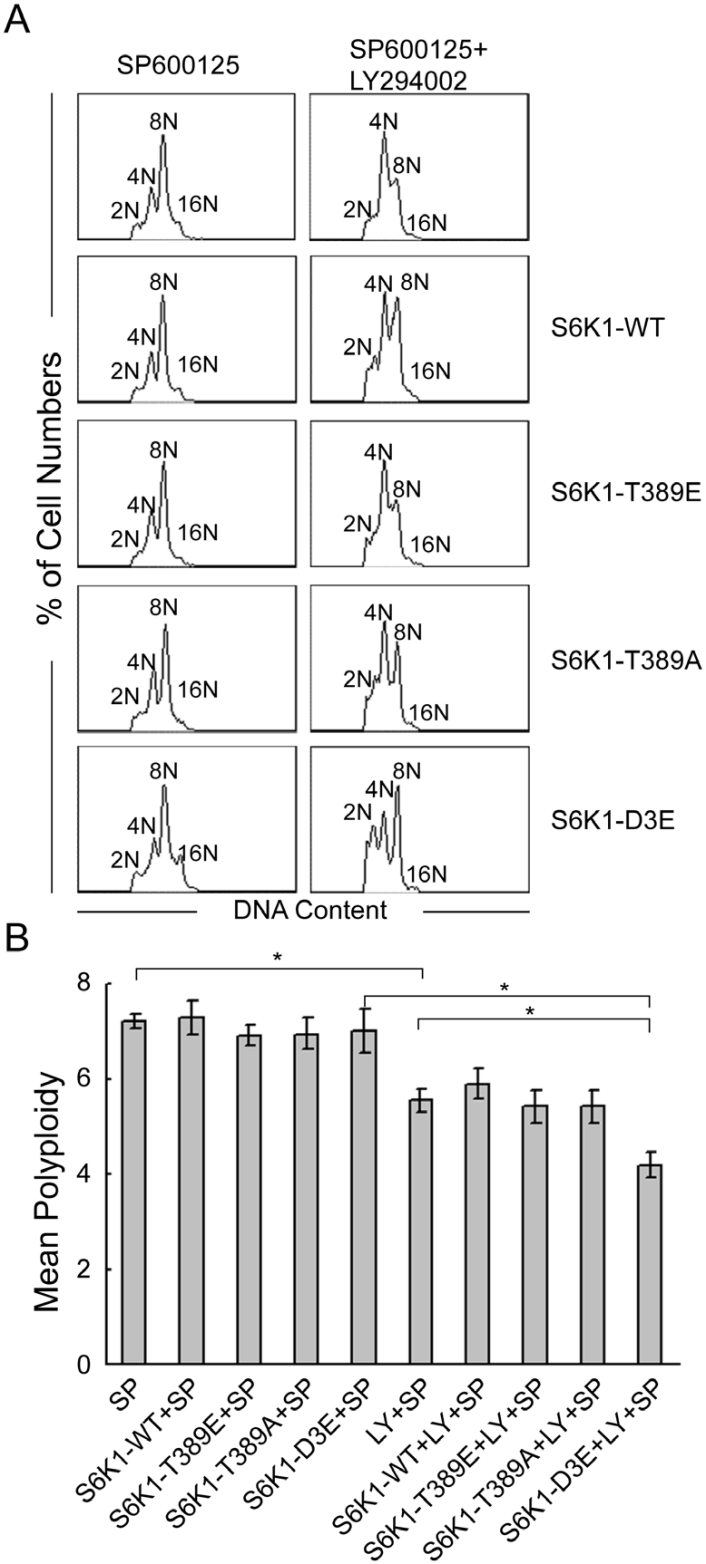
Effects of S6K1 mutant plasmids on SP600125-induced Dami cells. Dami cells were transfected with or without S6K1-WT, S6K1-T389E, S6K1-T389A and S6K1-D3E and cultured overnight. The cells were then induced with SP600125 for 72 hours after pretreatment with or without LY294002 for 1 hour. (A) Representative DNA histograms of Dami cells in each condition. (B) The data are presented as the mean±SEM of the level of polyploidy and were obtained from 4 separate experiments; *p<0.05.

We previously reported that myc-d/ED3E-pRK5 containing the mutations of Ser411 to Asp, Ser418 to Asp, Thr421 to Glu, Ser424 to Asp and Thr389 to Glu, which is rapamycin-resistant mutant, blocked the nocodazole-induced polyploidization of Dami cells [7]. In this study, we further found that the SP600125-induced polyploidization of Dami and CMK cells was accompanied by the phosphorylation of S6K1 at Thr421/Ser424 and dephosphorylation at Thr389 and that H-89 blocked the SP600125-induced polyploidization of Dami and CMK cells by concomitantly increasing the phosphorylation of S6K1 at Thr389 and decreasing the phosphorylation at Thr421/Ser424. Therefore, we constructed plasmids encoding mutated S6K1 using single site-directed mutagenesis or multi-site-directed mutagenesis targeting the C-terminal autoinhibitory pseudosubstrate domain and the hydrophobic motif of S6K1 (see table 1) to determine which domain of S6K1 was predominantly involved in the regulation of polyploidization. Dami and CMK cells were transfected with these plasmids and then induced with SP600125. Unexpectedly, none of these mutant plasmids had a significant effect on the SP600125-induced polyploidization of Dami cells (Fig. 6A and B) that were either untreated or pretreated with U0126 or PD184352 (data not shown). However, when the cells were pretreated with LY294002, S6K1-D3E reduced the mean ploidy of SP600125-induced Dami cells (Figure 6B). Similar results were obtained with CMK cells (data not shown). S6K1-D3E contains mutations of Ser411 to Asp, Ser418 to Asp, Thr421 to Glu and Ser424 to Asp. Given that the phosphorylation of S6K1 at Thr421/Ser424 alone may not be sufficient to mediate polyploidization (described above), these data suggest that the phosphorylation of the C-terminal autoinhibitory pseudosubstrate domain of S6K1 may mediate the SP600126-induced polyploidization of Dami and CMK cells synergistically with other signaling pathways, independent of the Akt pathway.

## Discussion

MKs are a fairly rare cell type: within normal human bone marrow, MKs represent only 1 in 10000 nucleated cells [34]. Therefore, four different cell lines with megakaryocytic properties were employed in this study. By treating two of these cell lines (Dami and CMK cells) with SP600125, we successfully established relatively synchronized cell models of polyploidy with polylobulated nuclei. The SP600125-induced polyploidization of these models is similar to the physiological polyploidization of primary MKs with a unique multilobulated nucleus. Furthermore, cyclin B1, cyclin D3, c-Myc, and survivin, which are involved in the regulation of polyploidization of MKs [17], [18], [19], [20], [21], [22], were upregulated during the polyploidization of Dami and CMK cells. Therefore, SP600125-induced polyploidization of Dami and CMK cells is a useful approach for investigating the mechanism of megakaryocytic polyploidization. However, SP600125 did not significantly induce the polyploidization of Meg-01 cells, and it significantly reduced the viability of HEL cells despite inducing their polyploidization. MK, which originate from hematopoietic stem cells, undergo an early common myeloid progenitor (early CMP) stage, a megakaryocyte/erythroid progenitor (MEP) stage, and a megakaryocyte progenitor (MKP) stage before finally becoming polyploid megakaryocytes [14]. During this developmental process, platelet-specific antigens, including CD41 and CD42, are expressed gradually. Dami cells have high levels of CD41a, CD42a and CD42b, and CMK cells have a high level of CD41a, indicating that these cells represent more differentiated megakaryocytic cell lines (Fig. S1). Meg-01 cells have a low level of CD41a and represent a less differentiated megakaryocytic cell line. Finally, HEL cells originated from a patient with erythroleukemia and they only expressed CD41a. Our results indicated that the more differentiated leukemic cell lines in the megakaryocytic lineage were able to be efficiently induced to become polyploid by SP600125.

We found that SP600125 induced the polyploidization of Dami and CMK cells by increasing the phosphorylation of S6K1 at Thr421/Ser424 in the C-terminus and decreasing the phosphorylation at Thr389 in the hydrophobic motif. This conclusion was confirmed by the following data: (1) S6K1 was phosphorylated at Thr421/Ser424 and dephosphorylated at Thr389 during the polyploidization of SP600125-induced Dami and CMK cells; (2) when the polyploidization of SP600125-induced Dami cells and CMK cells was blocked by H-89, the phosphorylation of S6K1 at Thr421/Ser424 was reduced and the phosphorylation at Thr389 was increased; and (3) docking studies and an S6 kinase activity assay showed that H-89 bound directly to S6K1 and inhibited its activity, indicating that it changed the phosphorylation state of S6K1 through direct binding. This result is partially consistent with our previous finding that S6K1 was involved in polyploidization through its phosphorylation at Thr421/Ser424 in a nocodazole-induced Dami cell model [7].

The eIF3-PIC (translation pre-initiation complex) serves as a dynamic scaffold for mTOR- and S6K1-mediated assembly of the translation initiation complex [35]. Interaction between S6K1 and eIF3-PIC is governed by the activating phosphorylation of S6K1 on Thr389 in the hydrophobic motif residue. However, in SP600125-induced polyploid Dami and CMK cells, S6K1 was phosphorylated at Thr421/Ser424 and dephosphorylated at Thr389. Moreover, H-89 decreased the phosphorylation of Thr421/Ser424 and increased the phosphorylation of Thr389 while blocking the polyploidization of SP600125-induced Dami and CMK cells. The phosphorylation of Thr389 is critical for the release of S6K1 from the eIF3 complex. This phosphorylation results in S6K1 dissociation and activation followed by the subsequent phosphorylation of S6 and eIF4B at Ser422, which is then recruited into eIF3-PIC in a phosphorylation-dependent manner [35]. It is not known whether the phosphorylation of S6K1 at Thr421/Ser424 and dephosphorylation at Thr389 of S6K1 leads to the release of S6K1 from the eIF3 complex. However, the phosphorylation of these sites leads to a conformational change in S6K1 [36] that may disrupt the binding between S6K1 and eIF3-PIC.

However, the effect of SP600125 on S6K1 in Meg-01 and HEL cells was different from that in CMK and Dami cells. Although SP600125 mainly increased tetraploidy of Meg-01 cells, it had no significant effect on the phosphorylation of S6K1. In contrast, SP600125 increased the phosphorylation of S6K1 at Thr389 without a concomitant increase in the phosphorylation of Thr421/Ser424 in HEL cells, which leads to an increase in polyploidy in these cells, although the effect of SP600125 on HEL cells was weaker than that on CMK and Dami cells. In addition, SP600125 did not change the protein levels of cyclin B1, cyclin D3, c-Myc, and survivin in Meg-01 cells, and it only induced the expression of cyclin B1 and survivin in HEL cells. Moreover, H-89 did not block the SP600125-induced polyploidization of Meg-01 cells, and it further increased the SP600125-induced polyploidization of HEL cells, although it increased the phosphorylation at Thr389 in both cell lines. Taken together, these data suggested that SP600125-induced polyploidization of leukemic cell lines with megakaryocytic features is cell-type specific, that the CMK and Dami cells are more differentiated than Meg-01 and HEL cells, and that phosphorylation at Thr421/Ser424 and dephosphorylation at Thr389 of S6K1 may play an important role in SP600125-induced polyploidization of CMK and Dami Cells. It is generally believed that the phosphorylation of S6K1 at Thr389 is associated with its kinase activity. However, a recent investigation showed that phosphorylation of S6K1 at Thr389 and its related function are not associated with the kinase activity towards S6 [37], [38]. The kinase inhibitor PF-4708671 has been shown to induce Thr389 phosphorylation but to block p70 S6K1 kinase activity [38]. Although PF-4708671 triggers the downregulation of S6K1 activity towards S6, it still induces the nuclear localization of this kinase, which was accompanied by a strong induction of S6K1 phosphorylation at Thr389 [37]. Because S6K1 has multiple phosphorylation sites, it is presumably believed that S6K1 in different phosphorylation states may function differently in different types of cells.

It was previously reported that serotonin-induced growth of pulmonary artery smooth muscle and 15(S)-hydroxyeicosatetraenoic acid-induced angiogenesis require the phosphorylation of S6K1 at Thr421/Ser424, which was markedly blocked by LY294002 [39], [40]. Proline-directed mitogen-regulated MAPKs phosphorylated Thr421/Ser424 [32], and U0126 blocked TPA or insulin-induced phosphorylation of S6K1 at Thr421/Ser424 in cardiocytes [41]. In this investigation, we found that SP600125 induced the phosphorylation of p44/42 MAPK at Thr202/Tyr204 and the polyploidization of Dami and CMK cells. Both PD184352 and U0126 markedly inhibited the phosphorylation of S6K1 at Thr421/Ser424. However, PD184352 did not inhibit the SP600125-induced polyploidization of Dami and CMK cells, although it abrogated the phosphorylation of p44/42 MAPK at Thr202/Tyr204. In contrast, U0126 inhibited the SP600125-induced polyploidization of Dami and CMK cells, but it did not abrogate the phosphorylation of p44/42 MAPK. Moreover, LY294002 also inhibited the phosphorylation of Thr421/Ser424 and blocked the SP600125-induced polyploidization of Dami and CMK cells, although it increased the phosphorylation of Akt. Notably, PD184352, LY294002 and U0126 did not increase the phosphorylation of S6K1 at Thr389. Therefore, the MAPK and PI3K/AKT pathways might not be involved in the polyploidization induced by SP600125 in Dami and CMK cells. Furthermore, the phosphorylation of S6K1 at Thr421/Ser424 alone may not be sufficient to mediate the polyploidization of Dami and CMK cells induced by SP600125, and other signaling pathways may be involved in this process.

It has been proposed that the stepwise activation of S6K1 via complex multi-site phosphorylation is initiated by the phosphorylation of four sites (Ser411, Ser418, Thr421 and Ser424) in the C-terminal pseudosubstrate domain, which induces a conformational change that enables access to the hydrophobic motif (HM) and T-loop sites. Although the phosphorylation of these four C-terminal sites contributes to S6K1 activation, it is not critical. The mutation of these four sites to alanine residues or the deletion of 101 amino acids from the C-terminus (-CT) modestly reduces S6K1 activation [33]. In this investigation, we found that S6K1-D3E, which contains mutations of Ser411 to Asp, Ser418 to Asp, Thr421 to Glu and Ser424 to Asp, further blocked the polyploidization of SP600125-induced Dami and CMK cells that had been partially blocked by pretreatment with LY294002, although S6K1-D3E alone had no effect on the polyploidization of SP600125-induced Dami and CMK cells. However, S6K1 either with a mutation of Thr389 to Glu (S6K1-T389E) or with a mutation of Thr389 to Ala (S6K1-T389A) had no effect on the SP600125-induced polyploidization of Dami and CMK cells with or without pretreatment with LY294002. Our previous study showed that overexpression of the kinase-dead form of S6K1 (myc-2BQ-pRK5, KD), which contains a mutation of Lys100 to Gln, in PMA-induced Dami cells increased polyploidy, whereas overexpression of the rapamycin-resistant form of S6K1 (myc-d/ED3E-pRK5, RR), which contains mutations of Ser411 to Asp, Ser418 to Asp, Thr421 to Glu, Ser424 to Asp and Thr389 to Glu and increases the basal activity of the kinase, did not [7]. Moreover, myc-d/ED3E-pRK5 blocked the nocodazole-induced polyploidization of Dami cells [7]. Considering that H-89 blocked the polyploidization of SP600125-induced Dami and CMK cells via the dephosphorylation of S6K1 at Thr421/Ser424 and the phosphorylation of S6K1 at Thr389 (described above), we propose that the SP600125-induced polyploidization of Dami and CMK cells requires the coordinated phosphorylation of the C-terminal pseudosubstrate domain and dephosphorylation of HM in S6K1. However, the S6K1 protein can be subdivided into several important domains that regulate its activity through complex multi-site phosphorylation [33]. Therefore, further study is needed to understand the molecular mechanism of S6K1 during polyploidization.

Noticeably, the cells used in this investigation are leukemic cell lines, which may not exactly mimic the polyploidization of primary MKs. Moreover, Drexler and co-authors suspected that the Dami cells may have been contaminated by HEL cells [42]. However, the Dami cells that we used are different from HEL cells, particularly with regard to differentiation and maturation, because most of the Dami cells (66.1%) in this study expressed CD41b (a maker of mature MKs). Meanwhile, SP600125 induced phosphorylation of the proteins that are involved in translation-related signal transduction (S6K1, 4E-BP1, or eIF4E) and increased the expression of cyclins (cyclin B1 and cyclin D3), c-Myc, and survivin, which are involved in polyploidization as well. Thus, SP600125-induced polyploidization of CMK and Dami cells most likely mimics the polyploidization in primary MKs, and the data obtained with the model can provide important clues about the mechanism of endomitosis in primary MKs.

In summary, we successfully established two relatively synchronized polyploid cell models. By using these models, we demonstrated that S6K1 is involved in promoting the SP600125-induced polyploidization of megakaryocytic cell lines through the coordinated phosphorylation of Thr421/Ser424 and dephosphorylation of Thr389 on S6K1 in a cell-type-specific manner.

## Supporting Information

Figure S1 **Phenotypic analysis of cell lines with properties of the megakaryocytic linage.** HEL, Dami, Meg-01 and CMK cells were labeled with anti-CD3, CD13, CD19, CD33, CD34, CD41a, CD42a, CD42b, CD71 and CD235a antibodies and analyzed with a Canto II Flow cytometer. Isotypic antibodies were used as a negative control. The histograms represent a typical experiment.
(TIF)

Figure S2 **Effect of SP600125 on proliferation, viability, polyploidy, and expression of cyclin B1, cyclin D3, c-Myc, and survivin, and the translation-related proteins of Meg-01 and HEL cells.** Related to Figure 1. Meg-01 and HEL cells were seeded at 2×10^5^/ml in RPMI 1640 medium containing 10% FBS and treated with SP600125 at different concentrations for different periods of time as indicated. Meg-01 and HEL cells treated with DMSO were used as a control. (A, B) The cell number and viability, presented as the mean±SEM values, were obtained from 3 separate experiments. (C) Representative DNA histograms of SP600125-induced Meg-01 and HEL cells analyzed with flow cytometry. (D) The morphology analysis was performed by Wright-Giemsa staining of each cytocentrifuged preparation of Meg-01 cells induced by SP600125 or nocodazole (original magnification, 1000×). Meg-01 and HEL cells treated with DMSO or with SP600125 were lysed, and equal amounts of protein were analyzed by western blot to determine the protein levels of cyclin B1, cyclin D3, c-Myc, and survivin (E). The phosphorylation and protein levels of S6K1, eIF4E and 4E-BP1 (F). β-actin was used as an internal control.
(TIF)

Figure S3 **The effect of H-89 on the polyploidization of SP600125-treated Meg-01 and HEL cells.** Related to Figure 2. Meg-01 and HEL cells were treated with SP600125 at 32 µM and 24 µM, respectively, for 72 hours after pretreatment with or without H-89 at 5 µM or 10 µM for 1 hour. Meg-01 and HEL cells treated with DMSO were used as a vehicle-treated control, and cells treated with H-89 alone were used as a pretreatment control. After incubation, the cells were fixed, stained with PI and analyzed with a flow cytometer to determine the DNA ploidy (A). The data are presented as the mean±SEM levels of polyploidy and were obtained from 4 separate experiments (B). All bar graphs depict means ± SD, *p<0.05, **p<0.01. The remaining cells were lysed, and equal amounts of protein were analyzed by western blotting for cyclin B1, cyclin D3, c-Myc, and survivin (C) and to determine the phosphorylation and protein levels of S6K1, eIF4E and 4E-BP1 (D). β-actin was used as an internal control.
(TIF)

Figure S4 **The binding mode of H-89 with phosphorylated S6K1.** Related to Figure 3. Docking studies were performed to evaluate the binding of H-89 to S6K1 using AutoDock 4.2 software. H-89 is predicted to bind into the hydrophobic cleft between the N- and C-terminal domains of phosphorylated S6K1 (PDB: 3A62).
(TIF)

Figure S5 **The effect of H-89 on the polyploidization of SP600125 treated Meg-01 cells independent of PKA.** Related to Figure 4. Meg-01 cells were treated with SP600125 at 32 µM for 72 hours after pretreatment with or without H-89 at increasing concentrations as indicated for 1 hour. Meg-01 cells treated with DMSO were used as a vehicle-treated control, and cells treated with H-89 alone were used as a pretreatment control. The cells were lysed, and equal amounts of protein were analyzed by western blotting for Phospho-PKA Substrate (RRXS*/T*), Phospho-(Ser/Thr) PKA Substrate, S6K1, phospho-S6K1 (Thr421/Ser424), and phospho-S6K1 (Thr389).
(TIF)
