# Supplementary figures and images for: Phosphorylation of Ribosomal Protein S6 Kinase 1 at Thr421/Ser424 and Dephosphorylation at Thr389 Regulates SP600125-Induced Polyploidization of Megakaryocytic Cell Lines
Source: PLoS One. 2014 Dec 8;9(12):e114389. doi: 10.1371/journal.pone.0114389 (PMC4259319; doi:10.1371/journal.pone.0114389)

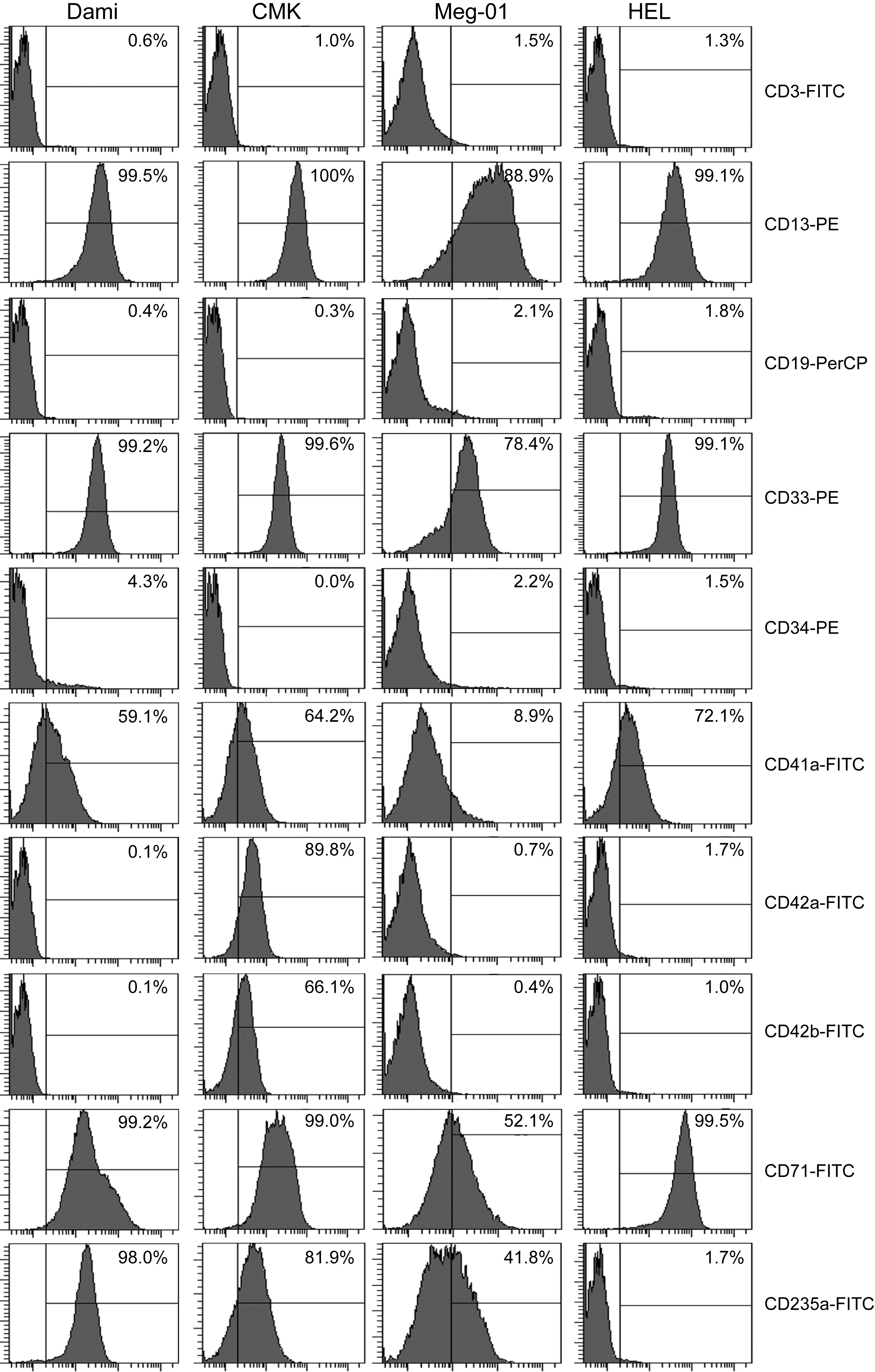

Supplement: Figure S1 — Phenotypic analysis of cell lines with properties of the megakaryocytic linage. HEL, Dami, Meg-01 and CMK cells were labeled with anti-CD3, CD13, CD19, CD33, CD34, CD41a, CD42a, CD42b, CD71 and CD235a antibodies and analyzed with a Canto II Flow cytometer. Isotypic antibodies were used as a negative control. The histograms represent a typical experiment. (TIF) [file pone.0114389.s001.tif]

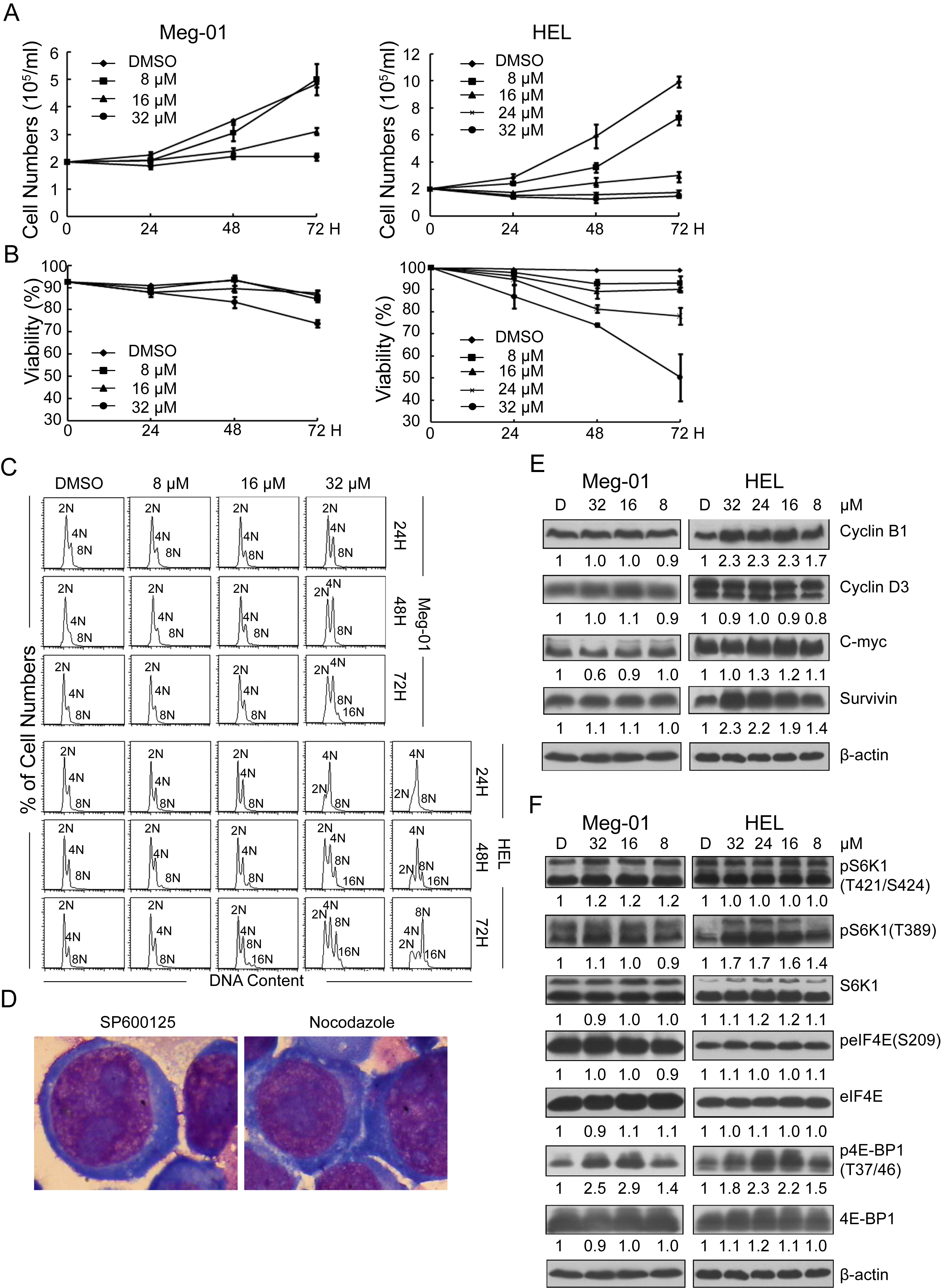

Supplement: Figure S2 — Effect of SP600125 on proliferation, viability, polyploidy, and expression of cyclin B1, cyclin D3, c-Myc, and survivin, and the translation-related proteins of Meg-01 and HEL cells. Related to Figure 1. Meg-01 and HEL cells were seeded at 2×10^5^/ml in RPMI 1640 medium containing 10% FBS and treated with SP600125 at different concentrations for different periods of time as indicated. Meg-01 and HEL cells treated with DMSO were used as a control. (A, B) The cell number and viability, presented as the mean±SEM values, were obtained from 3 separate experiments. (C) Representative DNA histograms of SP600125-induced Meg-01 and HEL cells analyzed with flow cytometry. (D) The morphology analysis was performed by Wright-Giemsa staining of each cytocentrifuged preparation of Meg-01 cells induced by SP600125 or nocodazole (original magnification, 1000×). Meg-01 and HEL cells treated with DMSO or with SP600125 were lysed, and equal amounts of protein were analyzed by western blot to determine the protein levels of cyclin B1, cyclin D3, c-Myc, and survivin (E). The phosphorylation and protein levels of S6K1, eIF4E and 4E-BP1 (F). β-actin was used as an internal control. (TIF) [file pone.0114389.s002.tif]

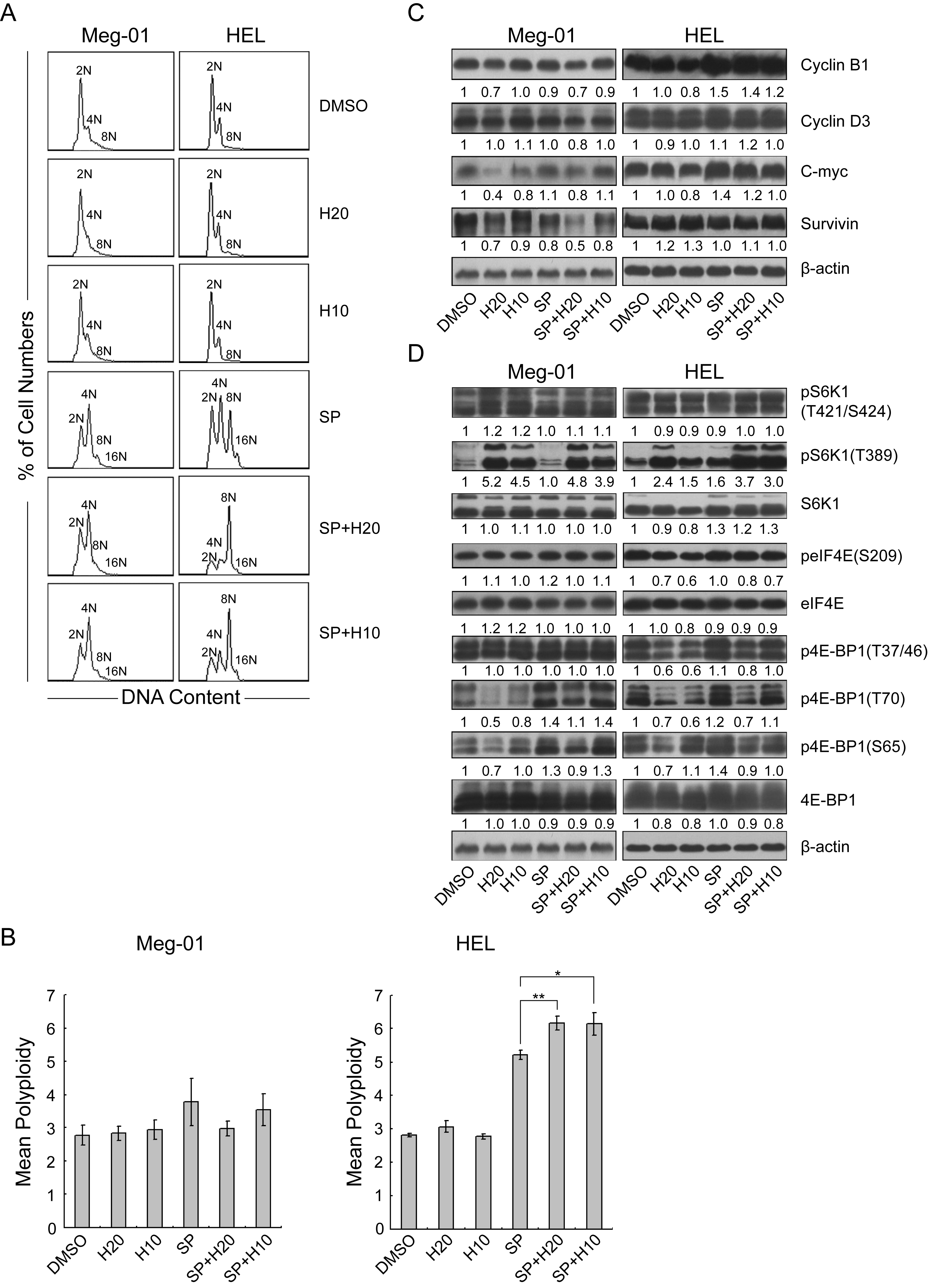

Supplement: Figure S3 — The effect of H-89 on the polyploidization of SP600125-treated Meg-01 and HEL cells. Related to Figure 2. Meg-01 and HEL cells were treated with SP600125 at 32 µM and 24 µM, respectively, for 72 hours after pretreatment with or without H-89 at 5 µM or 10 µM for 1 hour. Meg-01 and HEL cells treated with DMSO were used as a vehicle-treated control, and cells treated with H-89 alone were used as a pretreatment control. After incubation, the cells were fixed, stained with PI and analyzed with a flow cytometer to determine the DNA ploidy (A). The data are presented as the mean±SEM levels of polyploidy and were obtained from 4 separate experiments (B). All bar graphs depict means ± SD, *p<0.05, **p<0.01. The remaining cells were lysed, and equal amounts of protein were analyzed by western blotting for cyclin B1, cyclin D3, c-Myc, and survivin (C) and to determine the phosphorylation and protein levels of S6K1, eIF4E and 4E-BP1 (D). β-actin was used as an internal control. (TIF) [file pone.0114389.s003.tif]

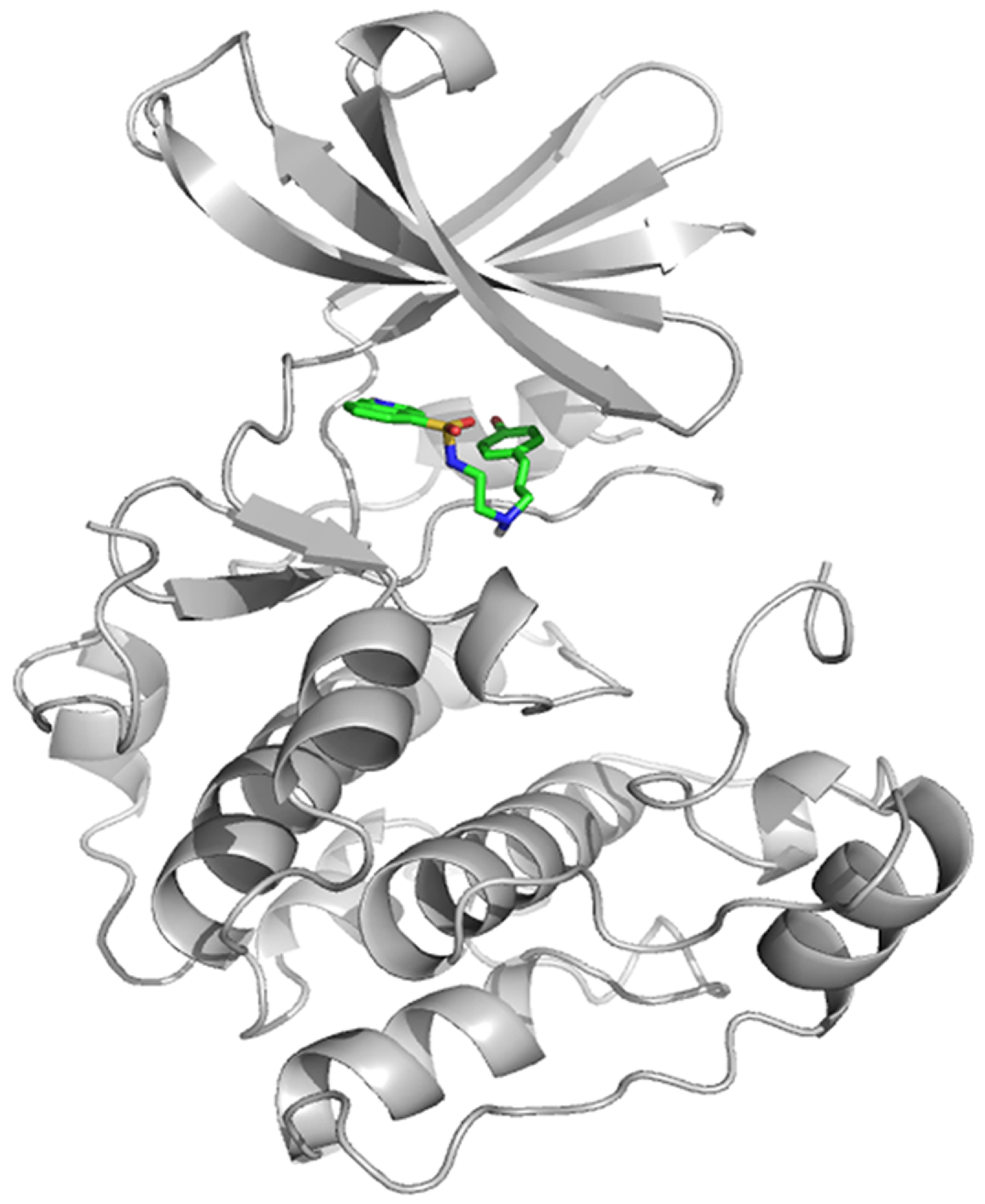

Supplement: Figure S4 — The binding mode of H-89 with phosphorylated S6K1. Related to Figure 3. Docking studies were performed to evaluate the binding of H-89 to S6K1 using AutoDock 4.2 software. H-89 is predicted to bind into the hydrophobic cleft between the N- and C-terminal domains of phosphorylated S6K1 (PDB: 3A62). (TIF) [file pone.0114389.s004.tif]

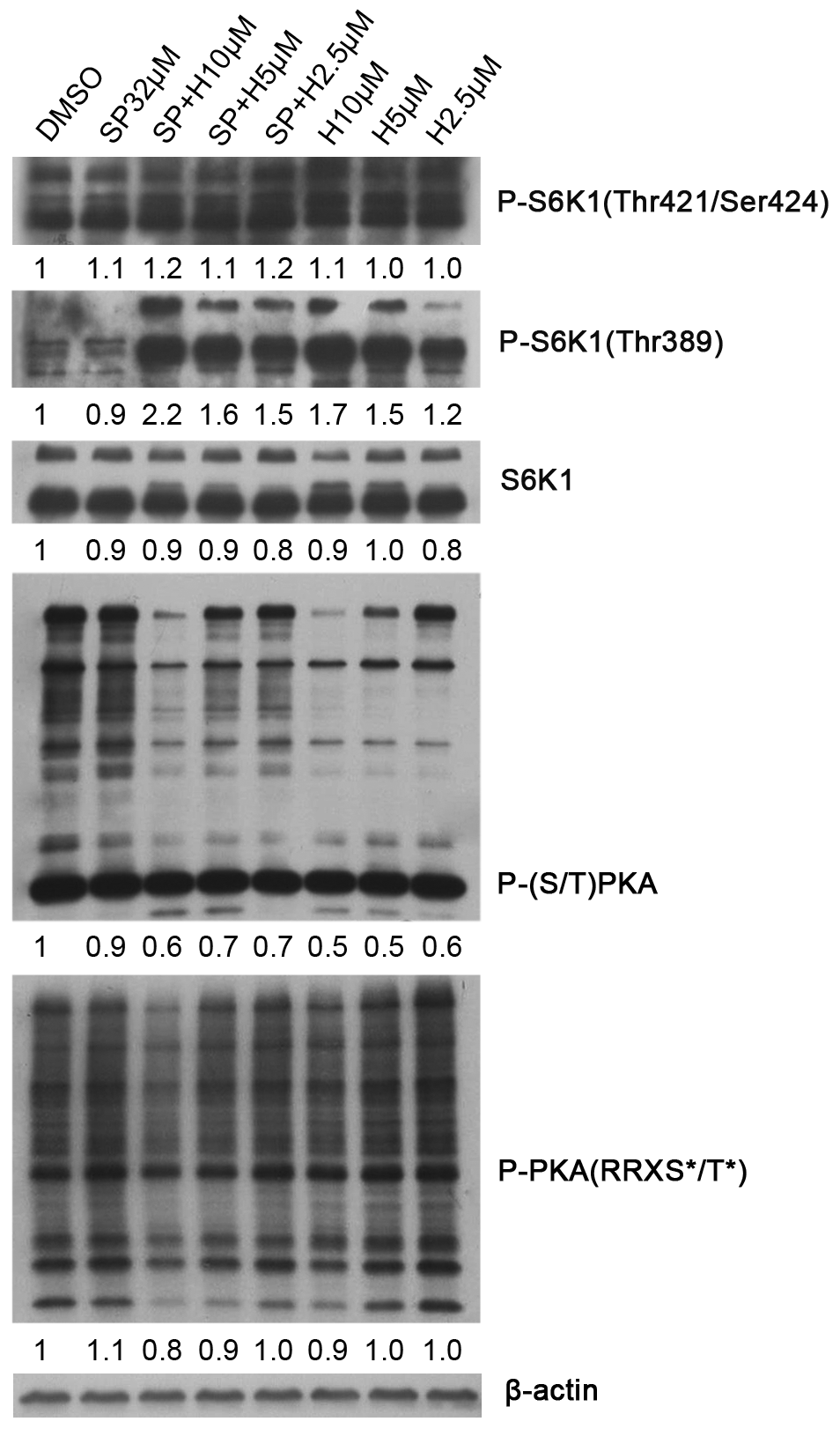

Supplement: Figure S5 — The effect of H-89 on the polyploidization of SP600125 treated Meg-01 cells independent of PKA. Related to Figure 4. Meg-01 cells were treated with SP600125 at 32 µM for 72 hours after pretreatment with or without H-89 at increasing concentrations as indicated for 1 hour. Meg-01 cells treated with DMSO were used as a vehicle-treated control, and cells treated with H-89 alone were used as a pretreatment control. The cells were lysed, and equal amounts of protein were analyzed by western blotting for Phospho-PKA Substrate (RRXS*/T*), Phospho-(Ser/Thr) PKA Substrate, S6K1, phospho-S6K1 (Thr421/Ser424), and phospho-S6K1 (Thr389). (TIF) [file pone.0114389.s005.tif]
